# Neurotoxicants Are in the Air: Convergence of Human, Animal, and *In Vitro* Studies on the Effects of Air Pollution on the Brain

**DOI:** 10.1155/2014/736385

**Published:** 2014-01-12

**Authors:** Lucio G. Costa, Toby B. Cole, Jacki Coburn, Yu-Chi Chang, Khoi Dao, Pamela Roque

**Affiliations:** ^1^Department of Environmental and Occupational Health Sciences, University of Washington, 4225 Roosevelt, Suite No. 100, Seattle, WA 98105, USA; ^2^Department of Neuroscience, University of Parma, Via Volturno 39, 43100 Parma, Italy; ^3^Center on Human Development and Disability, University of Washington, Seattle, WA 98195, USA

## Abstract

In addition to increased morbidity and mortality caused by respiratory and cardiovascular diseases, air pollution may also negatively affect the brain and contribute to central nervous system diseases. Air pollution is a mixture comprised of several components, of which ultrafine particulate matter (UFPM; <100 nm) is of much concern, as these particles can enter the circulation and distribute to most organs, including the brain. A major constituent of ambient UFPM is represented by traffic-related air pollution, mostly ascribed to diesel exhaust (DE). Human epidemiological studies and controlled animal studies have shown that exposure to air pollution may lead to neurotoxicity. In addition to a variety of behavioral abnormalities, two prominent effects caused by air pollution are oxidative stress and neuroinflammation, which are seen in both humans and animals and are confirmed by *in vitro* studies. Among factors which can affect neurotoxic outcomes, age is considered the most relevant. Human and animal studies suggest that air pollution (and DE) may cause developmental neurotoxicity and may contribute to the etiology of neurodevelopmental disorders, including autistic spectrum disorders. In addition, air pollution exposure has been associated with increased expression of markers of neurodegenerative disease pathologies.

## 1. Introduction

Air pollution is a mixture comprised of several components, including ambient particulate matter (PM), gases, organic compounds, and metals. The association between air pollution and morbidity and mortality caused by respiratory and cardiovascular diseases is well established [1–3]. Among air pollution components, PM is believed to be the most widespread threat and has been heavily implicated in disease [2, 4, 5]. PM is broadly characterized by aerodynamic diameter (e.g., PM_10_ and PM_2.5_, equivalent to <10 *μ*m and 2.5 *μ*m in diameter, respectively; [2]). Ultrafine particulate matter (UFPM; <100 nm) is of much concern, as these particles can more easily enter the circulation and distribute to various organs, including the brain [6, 7]. Of most relevance is also the fact that UFPM can gain access to the brain directly through the nasal olfactory mucosa, reaching first the olfactory bulb [7–11]. *In vitro *studies have shown that PM is cytotoxic and that toxicity is size-dependent, with the smaller UFPM being better able to enter the cells and exert toxic effects [12–15].

The populations of many countries (e.g., China, India, Middle East, and Central America) are commonly exposed for extended periods to relatively high levels (>100 *μ*g/m^3^) of PM [2], and such concentrations can be easily reached near trafficked roads and significantly exceeded in certain occupational settings [16]. Oxidative stress and inflammation are the two cardinal processes by which air pollution is believed to exert its peripheral toxicity [2, 17, 18]. PM has been shown to affect lung, cardiovascular, and nervous system functions by mechanisms that involve oxidative stress [19, 20], and oxidative damage has been shown to be a primary mechanism of PM toxicity [4, 19, 21]. For example, alterations in expression of some oxidative stress-related genes and other markers of oxidative stress have been shown in rodents following DE exposures [20, 22–25]. The same seems to be true with regard to the nervous system, as markers of oxidative stress and neuroinflammation are increased as a result of exposure to air pollution [7, 26].

Traffic-related air pollution is a major contributor to global air pollution, and diesel exhaust (DE) is its most important component [27]. DE contains more than 40 toxic air pollutants and is a major constituent of ambient PM, particularly of UFPM, and DE exposure is often utilized as a measure of traffic-related air pollution.

This commentary is intended to briefly summarize the main findings to date on the effects of air pollution (and more specifically of traffic-related air pollution) on the central nervous system, by analyzing and comparing findings from human epidemiological studies, controlled animal studies, and *in vitro* experiments. Particular attention is devoted to age as a potential susceptibility factor, as air pollution may play an etiological role in neurodevelopmental and neurodegenerative disorders. There appears to be a significant convergence of findings between humans and animals, pointing to similar alterations in the central nervous system (CNS) and to common mechanisms involving increased oxidative stress and neuroinflammation. Limited *in vitro* studies support these *in vivo* findings and should be expanded, to better define underlying mechanisms. A number of additional issues that warrant further investigations (e.g., role of gender, genetic susceptibility) are also discussed.

## 2. Neurotoxic Effects of Air Pollution in Humans

In recent years, evidence has been slowly accumulating suggesting that air pollution may negatively affect the CNS and contribute to CNS diseases [7, 26, 28–30]. Human epidemiological studies have shown that elevated air pollution is associated with decreased cognitive function in children, adults, and the elderly [29, 31–37]. Olfactory dysfunction, auditory deficits, depressive symptoms, and other adverse neuropsychological effects have also been reported [32, 35, 38, 39]. Few studies have examined neurotoxic effects following controlled exposure of humans to DE; for example, acute exposure to 300 *μ*g/m^3^ DE has been shown to cause airway inflammation and to induce EEG changes [27, 40].

Young as well as aging individuals appear to be particularly susceptible to air pollution-induced neurotoxicity [31–34, 36, 37, 41–43]. In case of pre- or postnatal exposures, this might cause or contribute to developmental disabilities and behavioral abnormalities, while during aging such exposures may contribute to neurodegenerative diseases. Indeed, increased incidences of neurodegenerative disease pathologies, namely, increased beta-amyloid 42, hyper-phosphorylated tau, and increased alpha-synuclein, have been found [41, 43–45], suggesting that air pollution may contribute to the etiopathogenesis of neurodegenerative diseases. Primary mechanisms of air pollution neurotoxicity appear to be related to oxidative stress and neuroinflammation, which are also involved in the etiopathology of various neurodegenerative diseases [46–49].

In addition to a series of animal studies (discussed below), human studies are also suggestive of developmental neurotoxicity of air pollution. Studies in Mexico City revealed elevated levels of neuroinflammatory markers in the brains of children exposed to high levels of air pollution [31, 43], as well as cognitive deficits [32]. In a recent comparison of children (average age 12 years) exposed to either high or low air pollution in various Mexican locations, the former were found to have higher levels of proinflammatory markers in the cerebrospinal fluid and in serum [50].

Newman et al. [51] reported hyperactivity in 7-year-old children associated with early life exposure to traffic related air pollution. Two studies by Volk et al. [52, 53] found that residential proximity to freeways and gestational and early life exposure to traffic-related air pollution were associated with autism (OR = 1.86; 95% CI 1.04–3.45). This was confirmed by another recent study in which perinatal DE exposure was significantly associated with autism spectrum disorders (ASD), particularly in boys [54]. The latter findings are particularly of interest, as there is increasing evidence indicating that children with ASD have higher levels of oxidative stress [55–57], as well as increased neuroinflammation and systemic inflammation [58–60]. As noted earlier, these are the typical effects found in individuals exposed to severe air pollution. The etiology of ASD is unknown, though ASD have a hereditary component [61, 62]. A number of candidate susceptibility genes for ASD have been identified, but no single anomaly appears to predominate, though the total fraction of ASD attributable to genetic inheritance may be about 30–40% [62]. Thus, environmental factors are increasingly suspected as playing a pivotal role in the etiology of ASD, most likely when affecting susceptible individuals [62–64]. Using mouse models of ASD to study the neurotoxicology of gene-environment interactions may be a promising novel avenue to investigate a potential role of air pollution in the etiology of ASD [65].

In summary, the available evidence in humans, albeit limited, is highly suggestive that exposure to high levels of air pollution can negatively affect the CNS and perhaps contribute to neurodevelopmental and/or neurodegenerative diseases. Furthermore, as indicated, markers of oxidative stress, neuroinflammation, and neurodegeneration are increased in postmortem human brain samples and in other accessible tissues, similarly to what is observed in animals.

## 3. Neurotoxic Effects of Air Pollution in Animals and *In Vitro* Studies

Animal observations and controlled studies confirm and expand the observations in humans, and limited *in vitro* evidence is suggestive of potential mechanisms. For example, in a postmortem study in Mexico City, similar neuropathological lesions in the prefrontal cortex were observed in both children and dogs [31]. Dogs exposed to Mexico City air pollution presented evidence of chronic inflammation, neurodegeneration, and DNA damage in various brain regions [28, 66]. In mice exposed to concentrated PM, neuroinflammation was seen in the brain, as evidenced by increased levels of various cytokines and of NF-*κ*B [67]. The latter result was also found in ApoE^−/−^ mice exposed to UFPM, together with increases in mitogen-activated kinase pathways and of GFAP (glial fibrillary acidic protein) [68]. Further studies in ApoE^−/−^ mice, which are known to be more prone to oxidative damage, have shown that subchronic exposure to concentrated ambient particles causes degeneration of dopaminergic neurons in the substantia nigra, as a result of oxidative stress [69]. Increases in proinflammatory cytokines (e.g., IL-1*β*, IL-6) have been found in the brains of mice exposed to traffic in a highway tunnel [70].

Several animal studies have involved controlled exposures to DE. Exposure of adult mice to DE has been reported to alter locomotor activity, spatial learning and memory, and novel object recognition ability [71–73]. Nasal instillation of nanoparticle-rich DE alters emotional behavior and learning capability in rats [74]. Biochemical and molecular studies have evidenced that some prominent effects of DE exposure on the CNS are oxidative stress and neuroinflammation [14, 19, 75]. Prolonged exposure of rats to DE was found to increase several proinflammatory cytokines in different regions of the brain, including the striatum [76]. A short-term exposure of rats to high levels of DE found similar induction of proinflammatory cytokines and other enzymes in brain regions and in lung [23]. Neuroinflammation may result from systemic inflammation which may affect the CNS through circulating cytokines or by direct entry of UFPM into the brain and activation of microglia [26]. Markers of neuroinflammation have been found to be increased upon long-term air pollution exposure in young adults at autopsy [43] and in rodents following DE exposure [16, 45, 76].

An *in vitro* study showed that DE particles can activate microglia, and microglia-derived oxidant species caused the demise of dopaminergic neurons [12]. Thus,* in vitro* evidence supports the *in vivo* findings of a role of microglia activation, oxidative stress, and neuroinflammation in DE neurotoxicity. DE particles have also been shown to cause oxidative stress and to increase inflammatory cytokines in brain capillaries *in vitro*, suggesting that DE particle may directly affect the blood-brain barrier [22]. Another recent *in vitro* study, in mouse hippocampal slices, found that DE nanoparticles altered glutamatergic neurotransmission and expression of NMDA receptors in the CA1 region [77], which might have relevance to the behavioral impact reported above.

There is also initial evidence that exposure to DE may cause developmental neurotoxicity in experimental animals [78]. *In utero* exposure to high levels of DE (1.0 mg/m^3^) caused alterations in motor activity, alterations in motor activity, motor coordination, and impulsive behavior in male mice, with concomitant alterations in dopamine and serotonin neurochemistry [79–81]. In another study, early postnatal exposure of mice to concentrated ambient PM has been reported to cause subtle behavioral changes (enhanced bias towards immediate rewards) without alteration in locomotor activity [82], while depression-like responses were found in mice exposed prenatally to urban air nanoparticles [83]. Additional studies showed that prenatal DE exposures of mice caused altered locomotor activity and spatial learning and memory, changes in gene expression (including X-chromosome inactivation factor), changes in neuronal differentiation, neuro-inflammation, and oxidative damage [71, 72, 80, 83–87]. Of interest is that the effects of prenatal exposure to DE on gene expression in the olfactory bulb were nullified by rearing the mouse pups with environmental enrichment [87], suggesting the possibility of therapeutic behavioral interventions in heavily exposed children. Prenatal DE exposure predisposes offspring to weight gain and to insulin resistance and induces neuroinflammation [88]. A recent study found that prenatal and early life exposure of mice to DE is associated with the presence of a number of behaviors similar to those in humans with ASD. These included higher levels of locomotor activity, elevated levels of self-grooming, and increased rearings [89].

In summary, examination of animals exposed to air pollution ambiently or in controlled experiments reveals the same pattern of neurotoxic effects (increased markers of oxidative stress and of neuroinflammation, age-related susceptibility) as in humans, suggesting that animal studies would be useful predictors of human outcomes. *In vitro* studies, albeit very limited so far, should be increased, as they should allow better definition of the exact mechanism(s) underlying the observed CNS damage.

## 4. Questions and Research Needs

Air pollution ranks among the top ten leading risk factors for mortality, possibly responsible for over 3 million deaths each year [7]. While respiratory and cardiovascular morbidity and mortality have been long seen as a primary consequence of high air pollution exposure, evidence emerging in the past decade suggests that the CNS may also be a significant target. As discussed in this commentary and in other recent reviews [7, 30, 39], human epidemiological studies and animal experiments are suggesting similar patterns of CNS damage, involving oxidative stress and neuroinflammatory processes. As these alterations are believed to be involved in the etiopathologies of neurodevelopmental and neurodegenerative diseases, it becomes apparent that the contribution of air pollution to CNS disorders may be of great significance. The convergence of findings in humans and animals is of much importance, as it will allow rapid testing and verification of hypotheses, which can also be corroborated by *in vitro* approaches. A number of issues should be considered, most of which have only been partially addressed.

### 4.1. Mechanisms of UFPM Neurotoxicity: Beyond the Olfactory Bulb

It is well established that PM may enter the brain and affect the olfactory bulb and either travel to “more remote” regions or allow the damage to be transmitted to such regions [8–11]. For example, olfaction problems have been reported in individuals exposed to heavy air pollution [38]. Olfactory dysfunction is also an early, important symptom of neurodegenerative diseases, particularly of Parkinson's disease [90–93], in which damage to the olfactory bulb actually precedes neuropathology in the motor areas, such as substantia nigra and striatum [94]. There is the need to better understand the events and mechanisms that lead to an involvement of the distal brain regions in the neurotoxic effects of air pollution. In addition to dopaminergic areas, which have been shown to be affected in certain studies (e.g., [69]), cholinergic and glutamatergic areas, important for cognitive behaviors and involved in other neurodegenerative disorders, may be affected by air pollution and should be further investigated. In addition, novel paradigms of neurotoxic damage may also be explored. For example, adult neurogenesis is believed to be essential for cognitive processes [95, 96] and has been shown to be negatively affected by neuroinflammation [96, 97]. Whether air pollution has any effect on adult neurogenesis remains to be investigated.

### 4.2. Peripheral versus Direct Central Effects

While air pollution components may exert their deleterious effects directly on the CNS, the possibility and the extent of a peripheral contribution to the central effects should be further explored. High levels of circulating proinflammatory cytokines may negatively affect the CNS [26, 50], and the blood-brain barrier may represent an important site for air pollution neurotoxicity that has been little studied so far.

### 4.3. The Role of Gender in Susceptibility to Air Pollution Neurotoxicity

While age represents an important factor of susceptibility in air pollution-induced neurotoxicity, two additional factors, gender and genetic background, should also be considered [98, 99]. With regard to gender, we had recently proposed that the differential expression of the enzyme paraoxonase 2 (PON2) in brain between males and females may be responsible for a number of gender differences in neurotoxicity [100]. PON2 is a mitochondrial enzyme, capable of scavenging reactive oxygen species (ROS), thereby protecting cells from oxidative stress-induced toxicity [100, 101]. In all tissues, brain regions, CNS cell types, and in both mice and humans, PON2, which has also anti-inflammatory properties, is expressed at higher levels in females [100, 102]. These findings prompted us to test the hypothesis that male mice may be more susceptible to air pollution neurotoxicity than female animals. Preliminary findings, shown in Table 1, appear to support this hypothesis, as, upon exposure of mice to DE, a marker of lipid peroxidation (malondialdehyde) and a proinflammatory cytokine (tumor necrosis factor-alpha) were increased in the hippocampus and to a greater extent in male animals. As air pollution may be involved in the etiopathogenesis of neurodevelopmental and neurodegenerative diseases whose incidence is usually higher in males (e.g., [103]), gender differences in the effects of air pollution deserve further investigations.

### 4.4. Genetic Susceptibility to Air Pollution Neurotoxicity

As in several other fields, gene-environment interactions should be investigated, as genetic polymorphisms may modulate susceptibility to air pollution neurotoxicity. Given the prominent role of oxidative stress, genetically based differences in antioxidant enzymes may thus predispose certain individuals to significant air pollution neurotoxicity. Glutathione (GSH) is one of the most abundant cellular thiols and a major player in cellular defense against ROS, as it nonenzymatically scavenges both singlet oxygen and hydroxyl radicals and is used by glutathione peroxidases, glutathione transferases, and peroxiredoxin-6 to limit the levels of certain reactive aldehydes and peroxides within the cell. When ROS production exceeds the antioxidant defense capacity of the cell, oxidative stress ensues, leading to damage of DNA, proteins, and membrane lipids. The first and rate-limiting step in the synthesis of GSH is carried out by glutamate-cysteine ligase which consists of a catalytic subunit (GCLC) and a modifier, or regulatory, subunit (GCLM) [105]. GCLC alone provides catalytic activity; however, in the absence of GCLM, the ability of GCLC to synthesize GSH is drastically reduced [106]. A relatively common C588T polymorphism has been discovered in the 5′-flanking region of the human *GCLM* gene, with individuals carrying the T allele having lower promoter activity in a luciferase reporter gene assay in response to oxidants and significantly lower plasma GSH levels [107]. Individuals with *GCLM* polymorphisms, or with other mutations leading to decreased GSH levels [106], would thus be expected to display an enhanced sensitivity to the adverse effects of environmental chemicals that elicit oxidative stress. *Gclm* null mice (*Gclm* ^−/−^) have very low GSH levels in all tissues including the brain [108] but may upregulate alternative antioxidant pathways, while *Gclm* heterozygous mice (*Gclm* ^+/−^) have only moderate reductions in GSH and may more closely resemble the human *GCLM* polymorphism. A preliminary testing of the hypothesis is shown in Table 2. Male mice exposed to diesel exhaust displayed the expected increase in markers of oxidative stress and neuroinflammation; of interest is that the effects were more pronounced in *Gclm* ^−/−^ and in *Gclm* ^+/−^ mice (Table 2). These results substantiate previous findings of enhanced lung inflammation in *Gclm* ^+/−^ mice compared to wild-type mice, upon exposure to DE [24]. Given the abundance of genetic polymorphisms of enzymes involved in oxidative stress [106, 109], this avenue of investigation warrants further research.

## 5. Conclusion

While considering potential sources of compounds that may contribute to the etiology of neurodevelopmental and neurodegenerative diseases, diet has often been considered as a primary vehicle of exposure. Indeed, many known neurotoxicants and developmental neurotoxicants are food contaminants. Yet, the air we breathe seems a logical potential source of chemicals which may exert neurotoxicity, though attention has been limited for several decades only to effects on the respiratory system and more recently on the cardiovascular system. Evidence has been accumulating during the past decade providing strong suggestions that exposure to high levels of high pollution, very common in many cities all around the world, is associated with damage to the CNS. Human and animal studies have evidenced a series of common adverse effects of air pollution (PM in particular), with oxidative stress and neuroinflammation emerging as the hallmark effects. A variety of behavioral alterations has also been reported, together with changes in some neurotransmitter systems. These and other aspects of air pollution neurotoxicity need to be further investigated. Particularly troublesome is the suggestion that air pollution may contribute to the etiopathology of neurodevelopmental and neurodegenerative diseases (e.g., autism or dementia) whose incidence is increasing in the global populations. Needless to say that, in addition to further biomedical research to augment our understanding of the effects and mechanisms of air pollution on the CNS, measures should be taken to curtail emissions and exposures.

## Figures and Tables

**Table 1:** Gender differences in the effects of diesel exhaust.

	FA	DE
MDA (nmol/g)		
M	4.7 ± 0.2	13.3 ± 0.3**
F	2.2 ± 0.1^#^	4.2 ± 0.2^∗#^
TNF-*α* (pg/mL)		
M	1.4 ± 0.4	9.8 ± 1.9**
F	0.7 ± 0.1	1.7 ± 0.2^∗#^

Male and female mice were exposed for 6 h to diesel exhaust (DE, 250–300 *μ*g/m^3^) or filtered air (FA), and levels of malondialdehyde (MDA) and of tumor necrosis factor-alpha (TNF-*α*) were measured in the hippocampus, as markers of oxidative stress (lipid peroxidation) and neuroinflammation, respectively. Results indicate the mean (±SE) with *n* = 3. DE versus FA: *P < 0.05; **P < 0.01. M versus F: ^#^ *P* < 0.05 (two-way ANOVA followed by Bonferroni test for multiple comparisons) (from [104]; Costa et al., unpublished results).

**Table 2:** Genetic polymorphisms and the effects of diesel exhaust.

	*Gclm *	FA	DE
MDA (nmol/g)	+/+	5.7 ± 0.2	21.2 ± 0.3**
	−/−	5.2 ± 0.3	35.4 ± 0.3^∗∗,#^
	+/−	9.7 ± 1.0	45.0 ± 0.7^∗∗,#^

IL-1*β* (pg/mL)	+/+	12.7 ± 1.6	31.1 ± 6.1**
	−/−	7.2 ± 0.3	42.6 ± 2.4**
	+/−	18.6 ± 0.7	78.5 ± 4.3^∗∗,#^

Male mice were exposed for 6 h to diesel exhaust (DE, 250–300 *μ*g/m^3^) or filtered air (FA), and levels of malondialdehyde (MDA) and of interleukin 1-beta (IL-1*β*) were measured in the olfactory bulb, as markers of oxidative stress (lipid peroxidation) and neuroinflammation, respectively. Results are the mean (±SE) with *n* = 3. DE versus FA: **P < 0.01; *Gclm* ^+/+^ versus *Gclm* ^−/−^ or *Gclm* ^+/−^: ^#^ *P* < 0.05 (two-way ANOVA followed by Bonferroni test for multiple comparisons) (Costa et al., unpublished results).
